# Systematic review and meta-analysis of the impact of STEM education on students learning outcomes

**DOI:** 10.3389/fpsyg.2025.1579474

**Published:** 2025-08-13

**Authors:** Xin Cao, Honglei Lu, Qian Wu, Yen Hsu

**Affiliations:** ^1^School of Art and Design, Fuzhou University of International Studies and Trade, Fuzhou, China; ^2^The Graduate Institute of Design Science, Tatung University, Taipei, Taiwan

**Keywords:** learning outcomes, meta-analysis, STEM education, systematic review, moderating variables

## Abstract

**Introduction:**

STEM education’s impact on student learning outcomes is nuanced, with differentiated effects across outcome types and academic levels.

**Methods:**

This study uses meta-analysis to systematically analyse 66 experimental and quasi-experimental studies on STEM education published in international English journals from 2000–2024.

**Results:**

The study finds that: (1) Subgroup analysis showed that STEM education had the most significant impact on cognitive outcomes in high school (*d* = 0.58) and reduced heterogeneity (*I*^2^ = 62.1%), while the overall effect size was exploratory due to construct diversity. (2) Overall, STEM education has a moderate effect on students’ learning outcomes, but the overall moderate effect size masks these key differences. (3) The effect of STEM education on students’ learning outcomes is influenced by the moderating variables of sample size, academic level, subjects, experimental period and teaching method.

**Discussion:**

These findings highlight the need to tailor STEM interventions to outcome type and academic level, strengthening the integration of theory and practice in STEM education.

## 1 Introduction

STEM education is a brand new educational practice, and STEM education has rapidly received widespread attention from industry and academia since it was first proposed in 1986 (Ortiz-Revilla et al., 2022). The main goal of STEM education is to cultivate students’ spirit of innovation, creativity, and practical ability. Traditional learning outcomes have centred on ‘knowledge mastery’ in the cognitive domain (e.g., memory, understanding), but modern educational theory has expanded its objectives to include the affective domain and motor skills domain. For example, Piaget and Duckworth (1970) proposed constructivism, which lists ‘creation’ as the highest level in the cognitive domain, clearly identifying problem-solving and creativity as higher-order learning outcomes (Runco and Pritzker, 2020); Bandura (1986) proposed social cognitive theory, which incorporates beliefs and attitudes related to ‘self-efficacy’ into learning objectives and emphasises its driving role in cognitive behaviour. Sweller (1994) proposed the Cognitive Load Theory, which states that students’ ability to produce innovative results is considered an important indicator of the impact of STEM education on student learning outcomes (Capraro et al., 2016). This classification is not merely pragmatic, but theoretically driven, aiming to capture the multidimensional characteristics of STEM education’s impact. However, STEM education is a multidisciplinary and complex learning-oriented process, and its impact on student learning outcomes has to be verified with the help of rigorous experiments rather than simple experiences or subjective judgements (Considine, 2014). As a result, a number of experimental or quasi-experimental studies on STEM education have been conducted by researchers (Gülen, 2019; Khalil et al., 2023), exploring the relationship between STEM education and student learning outcomes, and arriving at three very different conclusions.

The first finding suggests that STEM education has a positive impact on student learning outcomes. For example, a study by found that STEM education had a positive impact on the science and maths achievement of fourth-grade students. Kurt and Benzer (2020) conducted a study with sixth-grade students and found that the experimental group that received STEM education scored higher in academic achievement than the control group that used a constructivist approach. Lin et al. (2019) conducted a quasi-experimental study with 10th grade students and found that students with STEM-based education performed better in programming and physics achievement and had a higher sense of self-efficacy in modelling. Sirajudin and Suratno (2021) used a sample of all the students in the biology education research programme at Khairun University and found that STEM can be used as an alternative method of learning biology, especially in improving students’ creative thinking skills.

The second finding confirms that STEM education only enhances some learning abilities. For example, Yildirim and Sidekli (2018) found that STEM education does not have a significant effect on primary school students’ creativity but positively promotes primary school students’ interest in learning and hands-on ability. Kurt and Benzer (2020) study showed that STEM education has a significant effect on primary school students’ self-efficacy, problem solving, group collaboration and communication skills, but had no positive effect on creativity.

A third finding comprehensively rejects the impact of STEM education on student learning outcomes. For example, found no statistically significant difference between the academic performance of students using T-STEM education and non-T-STEM education. Cervetti et al. (2012) conducted a study of 937 primary school students on STEM education with the themes of reading comprehension and scientific writing, and showed no significant change in primary school students’ learning outcomes did not change significantly. The study conducted by Townes (2016) similarly showed that STEM education did not significantly enhance learning outcome metrics such as attitude towards learning and creativity levels among high school students.

In summary, there is no consistent conclusion on the impact of STEM education on student learning outcomes. In fact, the learning outcomes of students based on STEM education are influenced by a variety of factors such as sample size, academic level, subjects, experimental period, and teaching method (Wahono et al., 2020).

Meta-analysis is a quantitative research method that analyses the results of multiple experiments on the same topic. It allows for the synthesis of existing studies and a more accurate and objective assessment of their corresponding metrics (Pigott and Polanin, 2020). Meta-analysis differs from traditional literature review methods in that it focuses on comparing the results of different studies and providing an overall effect size through the same criteria (Rosenthal and DiMatteo, 2001). Meta-analysis, as a combination of qualitative and quantitative analyses, is able to synthesise the commonalities between individual studies with inconsistent findings on the same research topic, and ultimately develop consistent, generalised, and more precise findings by integrating individual studies (Zhao et al., 2024). Due to the differing purposes of the tests included in the study, this research adopts a broad definition of learning outcomes to avoid publication bias caused by indicator limitations. According to meta-analysis methodology by Cohen (1992b), when original studies exhibit measurement heterogeneity, integrating multi-dimensional indicators better reflects the overall effect of the intervention. This study references Linnenbrink-Garcia et al.’s (2010) “three-dimensional learning outcomes model,” defining learning outcomes as a combination of cognitive acquisition, motivational development, and skill transfer. Self-efficacy (Schwarzer and Jerusalem, 1995) reflects learners’ psychological resource reserves, problem-solving ability (Jonassen, 2011) and creativity (Runco and Pritzker, 2020) reflect the application and innovation of knowledge, aiming to comprehensively capture the multidimensional effects of educational interventions.

Based on this, this study used meta-analysis to analyse 66 experimental and quasi-experimental studies on the effects of STEM education on student learning outcomes, to explore the effects of STEM education on student learning outcomes, and to further explore how student learning outcomes are affected by five moderating variables: sample size, academic level, subjects, experimental period and teaching method. These variables were identified as moderators in previous studies and formed the basis for the use of these moderating variables in this study (Coban et al., 2022; Wu et al., 2020; Yu, 2023). The learning outcomes in this study are not a single concept but follow the classic framework of ‘cognitive-affective-motor skills’ in educational measurement (Bloom et al., 1956), divided into cognitive ability, non-cognitive ability, and skill performance. Cognitive ability include academic performance and knowledge retention rates, which are measured using standardised tools(Younas et al., 2025). Non-cognitive ability encompass psychological traits such as self-efficacy (Bandura, 2006) and learning interest (Yildirim and Sidekli, 2018). Skill performance includes observable behavioural manifestations such as problem-solving ability and collaborative ability (Kurt and Benzer, 2020).

## 2 Materials and methods

### 2.1 Literature search

In this study, the keywords of STEM education and learning outcomes were used to conduct the literature search in the scientific databases Scopus, Web of Science, and Google Scholar in strict accordance with the guidelines of PRISMA, which has detailed process standards that can be applied to most other literature review types of studies. The keywords for STEM education are ‘STEM’, ‘STEM Education’, ‘STEM Teaching’, ‘STEM Learning’. The keywords for learning outcomes are ‘Learning Result’, ‘Study Outcomes’, ‘Learning Effect’, ‘Study Performance’, ‘Learning Effect’ and ‘Study Performance’. Use search formulas to combine keywords from each category with the Boolean operator OR, and then further connect each combination of keywords with the operator AND. For example, (STEM OR STEM Education OR …) AND (Learning Outcomes OR Learning Results OR …). A search of journal literature from the above mentioned scientific databases for the period 2000–May 2024 was conducted, and the selected literature were all from international refereed journals listed in SSCI, AHCI or SCI, and a total of 2,568 literature were found to meet the requirements, of which 568 literature were from Scopus, 763 literature from Web of Science, and 1,237 literature from Google Scholar.

### 2.2 Literature screening

Since not all of the retrieved literature meets the requirements, it is necessary to screen the literature, this study used Cohen’s Kappa consistency test to verify the reliability of each literature screening process, and established the following screening criteria: (1) the literature is experimental or quasi-experimental research, review articles and theoretical articles were excluded; (2) the literature should report the indicators of learning effects (e.g., academic performance or creativity), and articles with no learning effects were excluded; (3) there should be an experimental group and a control group, and literature without a control group was excluded; (4) Literature that provided sufficient data to be able to calculate the experimental effect size, literature that could not be calculated was excluded, e.g., data that included the Mean, SD, and N of the experimental and control groups; (5) Duplicates were excluded, if the same piece of literature was published in different journals, or in different formats, only one was selected.

**Figure 1:**
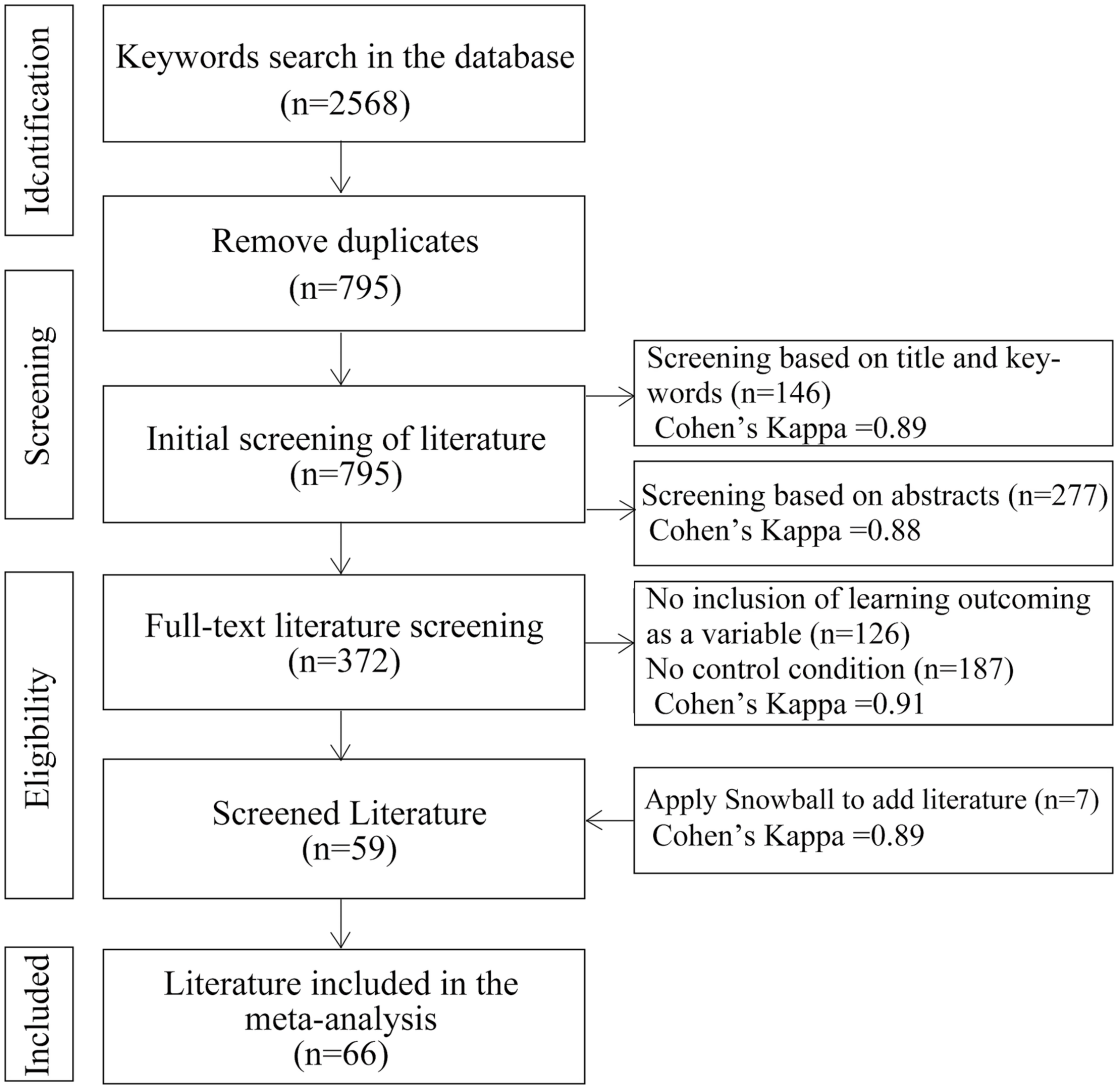
PRISMA process for literature screening.

The 2,568 literature data searched were imported into Endnote, and 795 literature were left after deleting duplicates, and 795 literature were left after completing the initial screening. Then the literature was double screened by two experts in related fields based on topics and abstracts, and Cohen’s Kappa coefficients were 0.89, 0.88, 0.91, and 0.89, respectively, which were all greater than 0.8 (almost perfect agreement), which proves that the screening results have good reliability (Borenstein et al., 2021), and 795 literature were screened to obtain 59 literature. The study applied the snowball method, which tracks citations forward and backward on top of this literature to find other relevant literature (Lim et al., 2018). Seven literatures were added using the ‘snowball’ method, resulting in 66 literatures as shown in Figure 1. The snowball method was chosen because it involves references cited in the selected literature (Wohlin, 2014). This approach not only benefits from checking the initial list of references only, but also complements it by checking the references cited in the literature (Suárez-Eiroa et al., 2019). The snowball method is a better method to extend systematic literature research than searching databases (Wohlin, 2014).

### 2.3 Literature coding

The impact of STEM education on student learning outcomes may be influenced by moderating variables such as sample size, academic level, subjects, experimental period and teaching method (Garzón and Acevedo, 2019; Zhang et al., 2020). This study is based on the meta-analysis framework proposed by Cooper et al. (2019) and employs a multi-dimensional hierarchical coding model to categorise moderator variables into three layers: contextual layer (sample size, academic level), intervention layer (subjects, experimental period, teaching method), and outcome layer (learning outcomes). In the sample size category, class sizes are categorised as small (1–50), medium (51–100), and large (>100) based on the recommendations proposed by Chingos (2013). In the academic level category, participants are categorised as primary school, secondary school, and university students based on Piaget’s stages of cognitive development (Diamond, 2013). In the subjects category, according to the framework of the National Research Council (2014), STEM corresponds to science (research focused on natural sciences), technology (research involving digital tools or robotics), engineering (research centred on design or manufacturing), and mathematics (research emphasising quantitative reasoning). In the teaching method category, based on the three-dimensional teaching model of STEM education, and corresponding to the knowledge integration model proposed by Bransford et al. (2013), it is divided into problem-oriented, project-oriented, and inquiry-oriented. Therefore, in addition to coding the overall learning outcomes and groups, it is also necessary to code the above moderator variables. The specific coding is shown in Table 1.

**Table 1:** Coding of moderator variables.

Moderator variables	Code
Sample size	a1 = 1–50, a2 = 51–100, a3= > 100
Academic level	b1 = Primary Schools, b2 = High Schools, b3 = Universities
Subjects	c1 = Technology, c2 = Engineering, c3 = Science, a4 = Mathematics
Experimental period	d1 = <1 weeks, d2 = 1–5 weeks, d3 = 5–10 weeks, d4 = > 10 weeks
Teaching method	e1 = Problem-orientated, e2 = Project-orientated, e3 = Inquiry-orientated

Two researchers with a major in education were selected for this study to content analyse 66 pieces of literature. First, training was provided on the meaning of the coding system and the coding methodology. Then, 10 randomly selected literature were pre-coded and inconsistent codes were explained and analysed so that a consistent understanding of the coding system could be reached. Finally, two researchers independently coded all the literature. After coding, the coding results of the two researchers were checked again and the reliability of the codes was calculated. For the inconsistent coding, the results of the discussion between the two researchers were selected. In this study, Cohen’s Kappa coefficient was used to calculate the consistency of the coding results, and the consistency coefficient was 0.88, which indicated that the coding results had good reliability.

### 2.4 Data analysis

In order to comprehensively explore the impact of STEM education on student learning outcomes, this study followed the analytical steps of Cooper et al. (2019) and used the Comprehensive Meta-Analysis (CMA) Version 3 software developed by Biostat to process and deeply profile the data. The meta-analysis study mainly used the fixed effects model and random effects model proposed by Borenstein et al. (2021). This study found that the relationship between STEM education and learning outcomes may be influenced by complex factors such as sample size, academic level, subjects, experimental period and teaching method. When different valid literature eigenvalues affect the results of meta-analysis, the random effects model should be chosen as the statistical model for meta-analysis, and the impact of STEM on students’ learning outcomes should be explored through publication bias test, heterogeneity test, overall effect value analysis and moderator variable analysis (Qiu et al., 2024).

In this study, the effect size calculation method proposed by Cohen (1988) was used as the combined effect value to assess the extent of the impact of STEM education on students’ learning outcomes (Cohen, 1988; Cooper et al., 2019), as shown in Equation 1. Where XE and XC represent the mean of learning performance of the experimental and control groups respectively, NE and NC represent the sample size of the experimental and control groups respectively, SE and SC represent the standard deviation of learning performance of the experimental and control groups respectively, and ES represents Cohen’s d.


(1) $$\mathrm{ES}=\frac{X_{E}-X_{C}}{\sqrt{\frac{(N_{E}-1)S_{E}2+(N_{C}-1)S_{C}2}{\left(N_{E}+N_{C}-2\right)}}}$$

## 3 Results

### 3.1 Publication bias test

Bias refers to the deviation of a study’s results or inferred values from their true values. In the field of social science research, research reporting bias is prevalent, and the test for publication bias is indispensable because only when the degree of publication bias is correctly evaluated can its impact on the results of meta-analysis be minimised (Thornton and Lee, 2000). Commonly used testing methods include the funnel plot method, Egger’s test, Begg’s test and loss of safety factor. Since Begg’s test is slightly more effective than Egger’s test and Begg’s is more sensitive to large samples (Pelletier et al., 1998), this study chose Begg’s test with funnel plot to detect publication bias.

**Figure 2:**
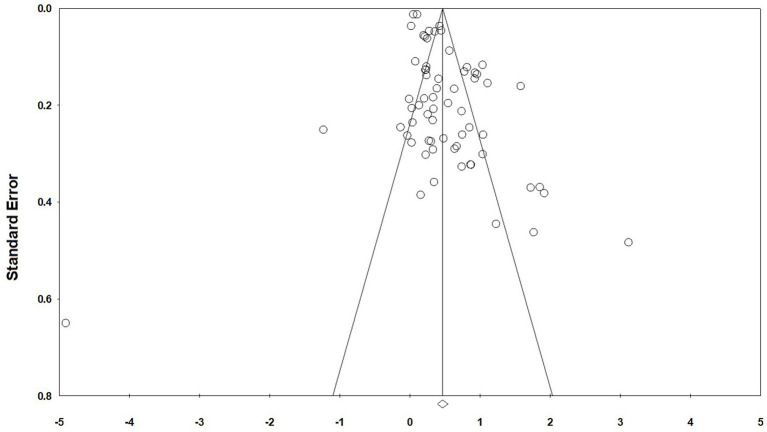
Funnel scatter graphic.

The funnel plot is characterised as being more intuitive, allowing the researcher to visually determine whether there is bias in the findings. The funnel plot centres on effect size (x-axis) and uses standard error (y-axis) as a measure of precision, illustrating the distribution characteristics of 66 studies. Ideally, the precision of effect size estimates improves with increasing sample size. Studies with small sample sizes, which have larger standard errors, are distributed at the bottom of the plot, while studies with large sample sizes are concentrated at the top, forming a symmetrical funnel shape (Petitti, 2001). If publication bias exists, the funnel plot will exhibit asymmetry. As shown in Figure 2, all effect points are distributed within the 95% confidence interval and are symmetrically distributed around the pooled effect value (0.5). The ratio of points on the left side (effect size < 0.5) to those on the right side (effect size > 0.5) is approximately 1:1, no obvious clustering or absence of points on either side is observed. The range of effect sizes in the small-sample studies at the bottom (−0.3 to 1.2) does not exceed the expected random error interval, and no clustering of points on the right side due to ‘positive result bias’ is observed. The results of Begg’s test show that *T* = −0.02 < 1.96, *p* = 0.80 > 0.05, which further indicates that there is no bias.

In this study, publication bias was also calculated using the Classic fail-safe N proposed by Rosenthal (1979), which assesses how many published studies are necessary for the total effect size of published studies to reach the level of non-significance. The measure is 5n + 10 (n refers to the number of papers included in the meta-analysis), and if the fail-safe N is much larger than 5 n + 10, it indicates that the effect size of the results of the unpublished studies does not have a significant impact on the overall effect size of the published studies. As shown in Table 2, the Classic fail-safe *N* result shows that the number of unpublished study results needed to reduce the overall effect size is 976, which is much greater than 340 (66 × 5 + 10).

**Table 2:** Results of classic fail-safe N.

Variable	Value
Z-value for observed studies	25.35
*p*-value for observed studies	0.00
Alpha	0.05
Tails	2.00
Z for alpha	1.95
Number of observed studies	66
Number of missing studies that would bring *p*-value to > alpha	976

In addition, this study used the JBI Critical Appraisal Checklist to assess the risk of bias in the 66 included studies. This tool comprises eight core criteria: studies with low bias met ≥6 criteria, studies with moderate bias met 4–5 criteria, and studies with high bias met ≤3 criteria. Quality assessment was conducted independently by two researchers, with consistency tested using Cohen’s Kappa coefficient (Kappa = 0.86, *p* < 0.001). Disagreements were resolved through discussion to reach consensus. The assessment results are shown in Table 3.

**Table 3:** Results of classic fail-safe N.

Research type	*N*	Low bias	medium bias	high bias	High bias reasons
Randomised experiment	28	18	8	2	Unreported allocation hidden (1 item), loss to follow-up rate > 20% (1 item)
Quasi-experiment	38	20	14	4	Baseline inconsistency (2 items), results measurement tools did not report reliability and validity (2 items)
Total	66	38	22	6	

Subgroup analysis by quality grade showed no statistically significant differences in effect sizes between studies with different risk of bias, *Chi*^2^ = 2.15, *p* = 0.34. The effect size for low-bias studies was 0.47, with *I*^2^ = 91.8%; for moderate-bias studies, the effect size was 0.45, with *I*^2^ = 93.2%; and for high-bias studies, the effect size was 0.41, with *I*^2^ = 89.5%. Sensitivity analysis showed that after excluding six high-risk studies, the overall effect size was 0.44, which was only slightly different from the original result (0.46), indicating that high-risk studies had limited impact on the overall conclusion.

### 3.2 Heterogeneity test

In order to investigate whether STEM education has a significant effect on students’ learning outcomes, this study used the heterogeneity test. The purpose of the heterogeneity test is to test whether all effect sizes come from the same whole (Petitti, 2001). A random effects model was used if there was heterogeneity between studies, and a fixed effects model was used if there was no heterogeneity (Rücker et al., 2008). Table 4 shows the results of the heterogeneity test and the overall distribution of effect sizes for the meta-analysis, with *Q* = 867.46, *p* < 0.0001 and *I*^2^ = 92.50%, indicating the presence of heterogeneity across samples. Where the statistic *I*^2^ reflects the proportion of the heterogeneity component in the overall effect value variation. The larger the *I*^2^ the greater the heterogeneity between the samples. From the results in the Table 4, it can be seen that *I*^2^ is 92.50%, which indicates that there is a large heterogeneity among the studies, so the random effects model should be chosen to eliminate the heterogeneity and the effect sizes should be combined.

**Table 4:** Heterogeneity test results.

Effect model	Combined effect size	95% CI		Two-tailed test	Heterogeneity test			
		Lower limit	Upper limit	*p*	*Q*	*I^2^*	*df*	*p*
Fixed effects model (FEM)	0.16	0.14	0.17	< 0.0001	867.46	92.50%	65	< 0.0001
Random effects model (REM)	0.46	0.38	0.54					

Since the concept of learning outcomes defined in this study has a relatively broad scope, it has led to high overall heterogeneity in the learning outcomes. Therefore, subgroup analysis was further conducted to categorize the learning outcomes based on the three aforementioned theories: cognitive ability (knowledge/academic performance), non-cognitive ability (attitude/self-efficacy), and skill performance (problem-solving/creativity). Each of these outcome types was assessed using its respective research framework—Bloom’s Taxonomy for measuring cognitive outcomes and Bandura’s Social Cognitive Theory for measuring self-efficacy. As shown in Table 5 subgroup analysis revealed that STEM education had a significant positive effect on all three constructs (*p* < 0.001), but the effect sizes differed (between-group *Chi*^2^ = 7.23, *p* = 0.027). The effect sizes for cognitive ability, non-cognitive ability, and skill performance were all moderate. Heterogeneity remains high, possibly due to statistical errors caused by the use of different measurement methods in the combined data.

**Table 5:** Subgroup analyses by outcome type.

Subgroup	*N*	Effect size	95% CI		Heterogeneity test *I*^2^ (%)	*Z*	*p*	Between-group effect size
			Lower limit	Upper limit				
Cognitive abilitiy	42	0.52	0.43	0.61	91.20	10.83	<0.001	*Chi*^2^ = 16.44, *p* < 0.001
Non-cognitive ability	16	0.38	0.25	0.51	87.60	5.72	<0.001	
Skill performance	8	0.41	0.22	0.60	89.30	4.26	<0.001	

Given the persistently high heterogeneity in learning outcomes, a more detailed exploration of heterogeneity was conducted by stratifying the studies according to outcome type (cognitive/non-cognitive/skill performance) and key contextual moderator variables (academic level). Since the heterogeneity associated with other moderator variables combined with outcome types did not decrease, this study did not further explore these combinations. As shown in Table 6, this study added a nested analysis table, which was stratified by outcome type (cognitive/non-cognitive/skills) and academic level (primary school/secondary school/university). The original overall heterogeneity *I*^2^ = 92.50%, while the *I*^2^ for cognitive outcomes at the high school level was 62.10%, significantly lower than the overall cognitive outcome of 91.20%, indicating that the effects of STEM education in the cognitive domain are more stable at the high school level. Among these, the highest combined effect size was observed for cognitive ability in high school (*d* = 0.58, *I*^2^ = 62.1, 95% CI = 0.47–0.69). Using the same stratification method, the combined effect size for non-cognitive ability in university students (*d* = 0.31, *I*^2^ = 89.1, 95% CI = 0.10–0.52) was the lowest.

**Table 6:** Nested heterogeneity analysis.

Outcome type	Academic level	*N*	Effect size	95% CI		Heterogeneity test *I*^2^ (%)	*Q*	*df*	*p*
				Lower limit	Upper limit				
Cognitive ability	Primary schools	10	0.39	0.25	0.53	89.60	86.42	9	<0.001
Cognitive ability	High schools	24	0.58	0.47	0.69	62.10	156.83	23	<0.001
Cognitive ability	Universities	8	0.45	0.29	0.61	88.10	58.73	7	<0.001
Non-cognitive ability	Primary schools	6	0.32	0.15	0.49	88.30	42.65	5	<0.001
Non-cognitive ability	High schools	7	0.42	0.26	0.58	87.70	48.92	6	<0.001
Non-cognitive ability	Universities	3	0.31	0.10	0.52	89.10	18.37	2	<0.001
Skill performance	Primary schools	3	0.35	0.12	0.58	88.20	16.89	2	<0.001
Skill performance	High schools	4	0.48	0.27	0.69	86.70	22.56	3	<0.001
Skill performance	Universities	1	0.39	0.11	0.67	-	-	-	<0.001

**Figure 3:**
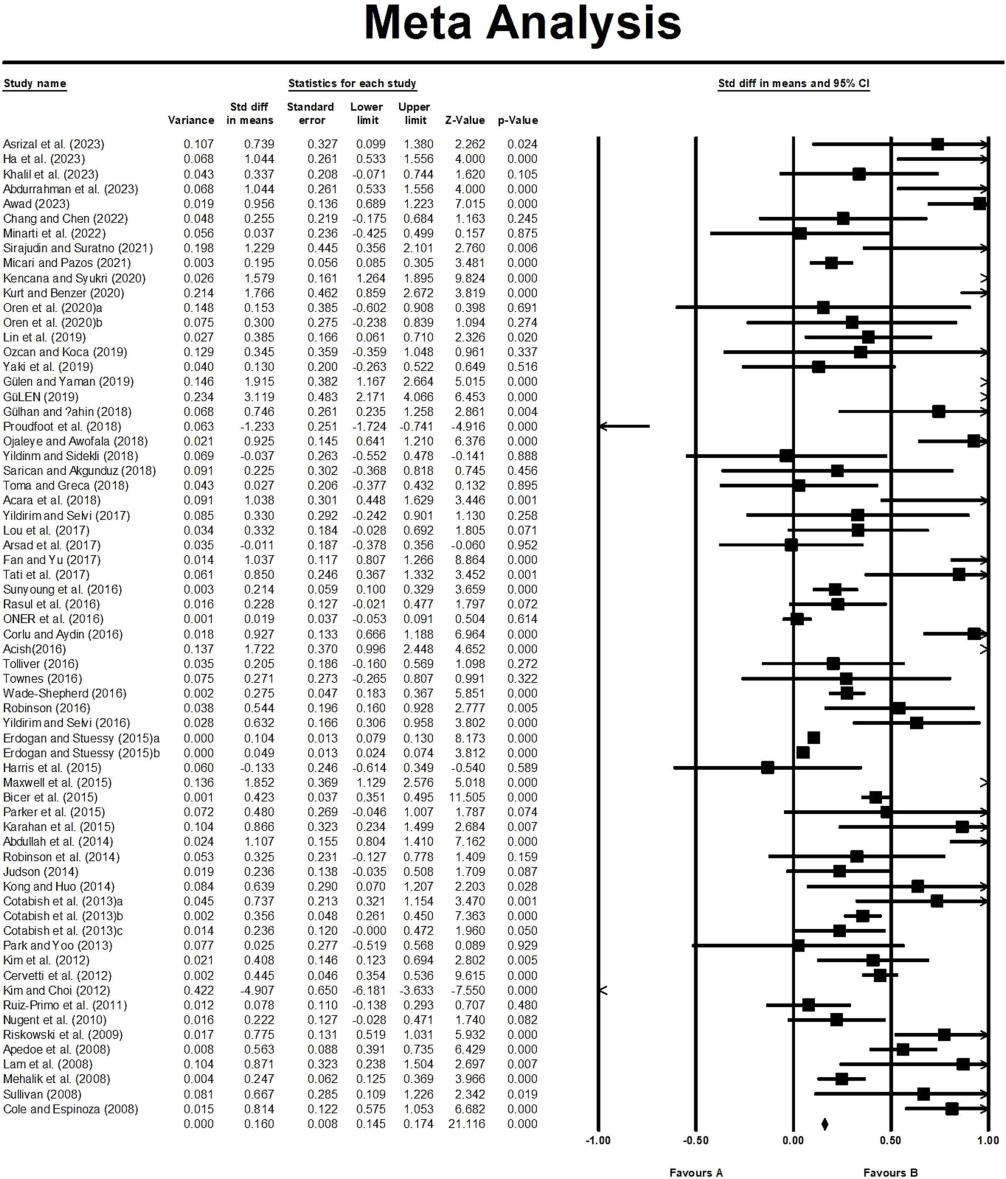
Forest map of the original studies.

In order to analyse the characteristics of the 66 studies in more depth, the effect values for each study were calculated in this study. Figure 3 presents the effect size, standard error, variance, lower limit, upper limit, *Z*-value, and *p*-value for each study. According to Cohen’s (1988) criteria for the classification of effect size 19 out of 66 studies had effect values of 0.8 and above, and the effect values reached statistical significance (*p* < 0.05); there were 32 studies with effect values of 0.2–0.8, of which 17 had statistically significant effect values, and there were 15 studies with effect values of less than 0.2, of which only 5 studies had statistically significant effect values. Thus, 41 studies showed that STEM education have a significant impact on the enhancement of learning outcomes (Cohen, 1988).

### 3.3 Sensitivity test

Sensitivity analyses are primarily used to examine outliers that may affect the overall effect size (Copas and Shi, 2000). In this study, One-Study Removal Analysis was used to examine the effect of extreme positive and negative effect sizes on the overall effect size. In this study, the range of 95% confidence intervals for the effect sizes after removing any of the studies was still 0.14–0.17 (Fixed Effects Model) and 0.38–0.54 (Random Effects Model), thus removing any of the studies did not affect the overall effect size. This is also a good indication that the meta-analyses produced in this study are very stable.

### 3.4 Effect size analysis

The results of the impact of STEM education on learning outcomes were obtained through CMA software analysis as shown in Table 4. From the random effects model, the combined effect size of the results of 66 studies was 0.46, with a 95% confidence interval of 0.38–0.54, and the combined effect size test *Z* = 11.91 (*p* < 0.0001) reached a statistically significant level. According to the theory of effect size analysis proposed by Cohen (1992a), when the effect size is around 0.2, the effect can be considered small; when the effect size is around 0.5, it is considered to have a moderate effect; and when the effect size is around 0.8, it is considered to have a significant effect. The overall effect size of 0.46 in this study indicates that STEM education has a moderate positive impact on students’ learning outcomes, a finding that proves that STEM education is conducive to improving students’ learning outcomes (Cohen, 1992a). This composite estimate integrates different constructs (cognitive, non-cognitive, skill performance) and should be interpreted with caution, as it masks significant differences in subgroup analyses.

### 3.5 Moderator variables analysis

#### 3.5.1 Sample size

In order to test the applicability of STEM education in different class sizes, the study divided the sample into small (1–50 students), medium (50–100 students), and large (more than 100 students) sizes based on class size. The specific analyses are shown in Table 7.

**Table 7:** Statistical analysis of mediator variable with different sample size.

Sample size	*N*	Effect size	95% CI		Heterogeneity test *I*^2^ (%)	*Z*	*p*	Between-group effect size
			Lower limit	Upper limit				
1–50	18	0.83	0.63	1.02	88.20	8.39	<0.001	*Chi*^2^ = 16.44, *p* < 0.001
50–100	16	0.37	0.20	0.53	64.93	4.47	<0.001	
>100	32	0.41	0.31	0.50	94.74	8.76	<0.001	
Total	66	0.52	0.27	0.77	92.50	4.15	<0.001	

As can be seen in Table 7, the between-group effect *Chi*^2^ = 16.44, *p* < 0.001, a statistically significant difference between the groups, suggesting that STEM education produced inconsistent levels of impact on students of different class sizes. The results of the data for small (effect value = 0.83, *Z* = 8.39, *p* < 0.001), medium (effect value = 0.37, *Z* = 4.47, *p* < 0.001), and large (effect value = 0.41, *Z* = 8.76, *p* < 0.001) sizes illustrate that the impact of STEM education has a positive contribution to the impact on small, medium, and large scale students, with the small scale students had the greatest influence and a significant influence, large-scale students had the second highest influence in terms of moderate impact, medium-scale students had the relatively least influence in terms of moderate impact, and small-scale, medium-scale, and large-scale students all reached the level of significant difference.

#### 3.5.2 Academic level

In order to test the applicability of STEM education at different academic levels, this study divided the sample into primary Schools, High Schools and Universities based on academic level, which was analysed as shown in Table 8.

**Table 8:** Statistical analysis of mediator variable with different academic level.

Academic level	*N*	Effect size	95% CI		Heterogeneity test *I*^2^ (%)	*Z*	*p*	Between-group effect size
			Lower limit	Upper limit				
Primary schools	22	0.33	0.19	0.46	88.75	4.68	<0.001	*Chi*^2^ = 6.38, *p* = 0.04
High Schools	36	0.54	0.44	0.64	93.40	10.61	<0.001	
Universities	8	0.42	0.21	0.62	87.52	4.05	<0.001	
Total	66	0.44	0.28	0.59	92.50	5.44	<0.001	

As can be seen in Table 8, the between-group effect *Chi*^2^ = 6.38, *p* = 0.04, a statistically significant difference between the groups, suggesting that STEM education does not have a consistent degree of impact on students at different academic levels. Primary schools (effect value = 0.33, *Z* = 4.68, *p* < 0.001), high schools (effect value = 0.54, *Z* = 10.61, *p* < 0.001), and universities (effect value = 0.42, *Z* = 4.05, *p* < 0.001) data results indicate that STEM education positively contributes to students’ learning outcomes impact are all positively contributing. Students in high schools had the greatest impact, followed by students in universities, and students in primary schools had the least impact, with all three academic levels achieving significant differences in student learning outcomes and had moderate impact.

#### 3.5.3 Subjects

In order to test the applicability of STEM education on different subjects, this study divided the sample into technology, engineering, science and mathematics based on the subjects, which were analysed as shown in Table 9.

**Table 9:** Statistical analysis of mediator variable with different subject.

Subject	*N*	Effect size	95% CI		Heterogeneity test *I*^2^ (%)	*Z*	*p*	Between-group effect size
			Lower limit	Upper limit				
Technology	14	0.52	0.33	0.70	85.81	5.54	<0.001	*Chi*^2^ = 5.57, *p* = 0.13
Engineering	7	0.73	0.45	1.00	90.83	5.22	<0.001	
Science	30	0.46	0.32	0.59	91.05	6.76	<0.001	
Mathematics	15	0.35	0.18	0.52	94.11	4.15	<0.001	
Total	66	0.49	0.34	0.63	92.50	6.70	<0.001	

From Table 9, the effect *Chi*^2^ = 5.57, *p* = 0.13, the difference between groups is not statistically significant, so it can be concluded that the effect of STEM education on the different subjects is relatively stable and does not produce significant differences. The effects of technology (effect value = 0.52, *Z* = 5.54, *p* < 0.001), engineering (effect value = 0.73, *Z* = 5.22, *p* < 0.001), science (effect value = 0.46 *Z* = 6.76, *p* < 0.001), and mathematics (effect value = 0.35, *Z* = 4.15, *p* < 0.001) the data results illustrate that the impact of STEM education on the learning outcomes of students in the four subjects has a positive facilitating effect. Students in engineering courses had the greatest impact on learning outcomes, followed by students in technology courses, then students in science courses, students in mathematics courses had the relatively least impact on learning outcomes, and students in all four subjects reached the level of significant difference in the enhancement of their learning outcomes and had moderate impact. Although the between-group differences across different subjects were not statistically significant, they accounted for 92.50% of the heterogeneity. There were no statistically significant differences between subjects.

#### 3.5.4 Experimental period

In accordance with the coding scheme, this study compared and analysed the impact of different experimental intervention cycles on the enhancement of student learning outcomes in STEM education, as shown in Table 10.

**Table 10:** Statistical analysis of mediator variable with different experimental period.

Experimental period	*N*	Effect size	95% CI		Heterogeneity test *I*^2^ (%)	*Z*	*p*	Between-group effect size
			Lower limit	Upper limit				
<1 weeks	9	0.63	0.41	0.86	78.88	5.61	<0.001	*Chi*^2^ = 4.22, *p* = 0.23
1–5 weeks	12	0.53	0.36	0.70	81.74	6.18	<0.001	
5–10 weeks	10	0.37	0.16	0.58	91.96	0.58	<0.001	
>10 weeks	35	0.42	0.32	0.52	93.35	8.54	<0.001	
Total	66	0.48	0.69	1.30	92.50	7.88	<0.001	

As can be seen from Table 10, the effect values of STEM education on the four different experimental period are 0.63, 0.53, 0.37 and 0.42, respectively, and the combined effect size is 0.48, with a two-tailed test (*p* < 0.001), which indicates that STEM education has a positive facilitating effect under these four experimental period, and in terms of the between-groups effect, *Chi*^2^ = 4.22, *p* = 0.23, which does not reach a significant level, so it can be concluded that the effect of STEM education on different experimental period is relatively stable and does not produce significant differences. Among them, the effect of <1 week is the most obvious, and the effect is 1–5 weeks, >10 weeks, 5–10 weeks in descending order, and all the experimental period have reached the level of significant difference and had moderate impact. There were no statistically significant differences between experimental periods.

#### 3.5.5 Teaching method

From the data in Table 11, the combined effect value of the three teaching methods was 0.46, *Z* = 9.24, *p* < 0.001, which shows that overall STEM has a moderately positive facilitating effect on the different teaching methods. However, in terms of between-group effects, *Chi*^2^ = 2.45, *p* = 0.29, the between-group effects were not significantly different. Therefore, there is no significant difference in the effect of different teaching methods on students’ learning. The effect value of problem-orientated = 0.54, *Z* = 7.70, project-orientated = 0.45, *Z* = 8.46, inquiry-orientated = 0.38 *Z* = 5.06, it can be seen that inquiry-orientated has the weakest degree of effect on students’ learning outcomes, problem-orientated has the best effect, followed by project-orientated, and all the three teaching methods are significantly different and had moderate impact. There were no statistically significant differences between teaching methods.

**Table 11:** Differences in the impact of different teaching method.

Teaching method	*N*	Effect size	95% CI		Heterogeneity test *I*^2^ (%)	*Z*	*p*	Between-group effect size
			Lower limit	Upper limit				
Problem-orientated	16	0.54	0.40	0.68	90.15	7.70	<0.001	*Chi*^2^ = 2.45, *p* = 0.29
Project-orientated	34	0.45	0.35	0.56	86.21	8.46	<0.001	
Inquiry-orientated	16	0.38	0.23	0.53	94.01	5.06	<0.001	
Total	66	0.46	0.36	0.56	92.50	9.24	<0.001	

## 4 Discussion

The increasing number of related studies in recent years indicates the growing academic interest in influencing student learning outcomes based on STEM education. This study adopts meta-analysis to systematically sort out and analyse the empirical studies on the impact of STEM education on students’ learning outcomes between 2000 and 2024, and verifies the differences in the effects of sample size, academic level, subjects, experimental period and teaching method on five moderating variables. The study found that:

1) First, STEM education has the most significant impact on cognitive ability in high school, with a combined effect size of 0.58. Second, STEM education has a moderate positive effect on students’ learning outcomes (cognitive ability, con-cognitive ability, skill performance). The results of this study are consistent with previous studies by other scholars (Lynch et al., 2019; D’Angelo et al., 2014; Mustafa et al., 2016). The mechanism of the impact of STEM education and students’ learning outcomes may be that STEM education integrates knowledge of disciplines that are separated and fragmented from each other, enabling students to understand the world according to a connected, dynamic, and systemic approach and develop innovative thinking in an iterative cycle of divergent and convergent thinking (Sgro et al., 2020). Meanwhile, STEM education guides students through a complete scientific evidence-seeking process through complex problems oriented to real situations, from which they gain intuitive experience of innovative practices, scientific rationality and self-management strategies (Pellas et al., 2017). All of these help to strengthen students’ construction and retention of knowledge, which in turn improves learning outcomes (Oprean and Balakrishnan, 2020). The overall effect size of learning outcomes (0.46) is a comprehensive indicator. However, the moderate overall effect size masks meaningful differences. For example, STEM education has a greater impact on cognitive outcomes (0.52), which is consistent with constructivism and may be related to its emphasis on knowledge integration, while its impact on non-cognitive outcomes is smaller (0.38), which may require longer-term intervention measures to develop. The overall effect size is not the final standard for measuring the impact of STEM education, but only a preliminary summary. Its explanatory value is limited by the integration of different measurement attributes (such as standardised tests in the cognitive domain and self-report scales in the self-efficacy domain) and differences in developmental trajectories. With the help of meta-analysis, the findings are inferential rather than causal, and the results of the data show that the effect of STEM education on students’ learning outcomes varies across the five moderating variables: sample size, academic level, subjects, experimental period, and teaching method. Therefore, the teaching effect of STEM is not absolute, and its promotion and popularisation need to be integrated with more research on the effectiveness of STEM teaching and in-depth analyses of the factors affecting the learning effect of STEM education on students.
2) Although subgroup analyses based on outcome type and academic level reduced heterogeneity (I^2^ values), the reduction in heterogeneity was most significant in cognitive ability in high school (I^2^ = 62.1%), residual heterogeneity remained at a high level, indicating the presence of unmeasured factors such as teachers’ STEM training, resource availability, or cultural attitudes toward STEM. These variables are likely to account for differences in effect sizes across different contexts, thereby limiting the generalisability of the pooled results. Qualitative comparisons of studies with extreme effect sizes revealed that studies with effect sizes greater than 0.8 (e.g., Khalil et al., 2023) typically employed problem-based approaches in high school engineering courses, while studies with effect sizes less than 0.2 (e.g., Townes, 2016) focused on non-cognitive outcomes in large university courses.3) The impact of STEM education on different learning outcomes exhibits a gradient difference, which is related to the measurement characteristics of the three learning outcome concepts and the core mechanisms of STEM education. Among these, the effect size for cognitive abilities is the highest, potentially due to STEM’s interdisciplinary integration characteristics, which directly strengthen the construction of knowledge networks by linking knowledge across disciplines such as mathematics and science (Maass et al., 2019). The sensitivity of standardised tests to such structured knowledge may also amplify effect sizes, as seen in mathematics achievement test. Effect sizes for skill performance rank second, as skills such as problem-solving and collaboration require assessment through complex tasks (Kurt and Benzer, 2020). However, some studies lack standardised task design, such as, whose assessment tools did not specify reliability and validity, may lead to fluctuations in effect sizes. Effect sizes for non-cognitive abilities are relatively the lowest, as improvements in constructs such as self-efficacy depend on long-term practice accumulation. However, 61% of the non-cognitive ability studies in this review had a duration of less than 10 weeks, and even in Yildirim and Sidekli (2018), whose test lasted more than 10 weeks, the effect size remained low, possibly because a stable impact had not yet been established. Additionally, the subjectivity of scale measurements (e.g., self-report bias in learning interest) may weaken effect size estimates.
3) In terms of sample size, all of the impacts of STEM education on student learning outcomes are moderately positively contributing. In particular, STEM education had the greatest impact on small-sized classes (1–50), followed by large-sized classes (> 100), and relatively the least impact on medium-sized classes (50–100). This finding is inconsistent with the results of, which may be due to the special nature of STEM education. STEM education emphasises interdisciplinary problem solving and practical innovation, and small class sizes provide conditions for frequent interaction (such as group brainstorming and engineering prototype iteration). Small-scale classes are suitable for writing-based learning and peer learning, based on the principle of peer effect on academic achievement, where peers are able to share information related to learning among themselves, and collaborative learning environments are effective in improving students’ learning outcomes (Ruengtam, 2018). This is consistent with the findings of small-sample studies by Aldilla et al. (2023) and Kurt and Benzer (2020), which demonstrate the reinforcing effect of collaborative learning environments on STEM outcomes. In contrast, in large-scale classroom versus medium-scale classroom environments, teaching and learning resources are diluted and teachers have fewer opportunities to encourage students to think, express and communicate (Abalde, 2014). At the same time, it may also be related to the characteristics of the STEM courses included in the study. For example, Micari and Pazos (2021) relied on problem-oriented teaching and online collaboration platforms to reduce the management costs of large-class teaching through standardised project processes, enabling students to still gain STEM practical experience within a structured framework. This model may be the key to the effectiveness of large-scale STEM education, which differs from the conclusion that the effectiveness of traditional large-class teaching inevitably declines. Therefore, it is important to consider the impact of class size on students in STEM education and focus on creating a collaborative and open learning environment for students. In summary, the class size effect in STEM education does not negate traditional educational research, but rather requires consideration of the special mechanisms formed by characteristics such as practice orientation and tool dependence. Future research needs to further distinguish the boundaries of the role of class size in different types of education (such as STEM vs. traditional subjects).
4) In terms of academic level, the impact of STEM education on students’ learning outcomes are all moderately positively contributing. In particular, STEM education has the most significant enhancement of learning outcomes for high school students, the second most significant impact on learning outcomes for university students, and the relatively smallest impact on learning outcomes for primary school students. Consistent with the results of, this may be due to the fact that students at the high school level have basically completed their subject matter knowledge base, and the intellectual skills required for problem solving and the cognitive strategies required for learning management are also available (Kong, 2014). At the same time, consistent with the self-determination theory proposed by Ryan and Deci (2000), high school students have stronger autonomy needs, and STEM education can better stimulate their intrinsic motivation by providing project-oriented support. High school is a critical period for self-identity formation and development, and the need for high school students to practice applying STEM knowledge across disciplines stimulates identity maintenance (Robinson, 2020). High school students are at an optimal stage of knowledge and mindfulness where learning ability occurs, thus enabling better performance in STEM education (Bowman et al., 2020). Comparatively, university students are less likely to produce more significant learning outcomes as their study habits are already developed and they have a clear purpose for learning and greater self-study skills (Du, 2020). According to Piaget’s theory of cognitive development (Huitt and Hummel, 2003), because the relative simplicity of what primary school students learn and their insufficient subject knowledge base, STEM education is not sufficient to attract their interest in learning (Scheerens, 2016), and more concrete experimental designs (such as < 1 week short-term interventions) are needed to reinforce learning outcomes.
5) In terms of subjects, the effects of STEM education on student learning outcomes are all moderately positively contributing. Among them, STEM education has the most significant contribution to the learning outcomes of students in the engineering programme. STEM education showed a positive impact across all disciplines, but the differences were not significant (*Chi*^2^ = 5.57, *p* = 0.13). This may be due to the small sample size in the engineering discipline (n = 7), resulting in insufficient statistical power. These results should be regarded as preliminary conclusions rather than evidence of no effect. Although there is no significant difference in learning outcomes among different subjects, this result can be used to encourage the promotion of interdisciplinary STEM education, and the flexibility of the cycle provides elasticity for teaching arrangements (Noor et al., 2022; Younas et al., 2022; Zhou et al., 2022).
6) In terms of experimental period, the effects of STEM education on student learning outcomes were all moderately positive. Among them, STEM education has the best effect in <1 weeks experimental period, followed by 1–5 weeks experimental period, then >10 weeks experimental period, and the smallest effect is 5–10 weeks experimental period. Effect sizes varied across periods but were non-significant (*Chi*^2^ = 4.22, *p* = 0.23). With inconsistent patterns (e.g., 5–10 weeks showing lower effects), these results are inconclusive and require replication with larger samples.
7) In terms of teaching method, STEM education has a moderately positive effect on students’ learning outcomes. Firstly, problem-orientated teaching method enhances students’ learning effect the best, followed by project-orientated teaching method’s effect on students’ learning effect, and lastly, inquiry-orientated teaching method’s effect on students’ learning effect, all teaching method achieved a significant effect. Effect sizes varied across teaching method but were non-significant (*Chi*^2^ = 2.45, *p* = 0.29). Non-significant moderator effects are reported for completeness but should not be interpreted as evidence of no effect. Their discussion is limited to methodological observations, as practical implications cannot be justified.

## 5 Conclusion

The main contribution of this study lies in revealing the differentiated effects of STEM education across different learning outcomes and contexts, rather than generalised effects. Key findings from the subgroup analysis include: STEM education has the most significant impact on cognitive outcomes, with high school students experiencing the greatest improvement, consistent with constructivist knowledge integration theory. Skill performance and non-cognitive learning outcomes are also positively influenced, but to a lesser extent, reflecting their unique measurement characteristics (e.g., non-cognitive learning outcomes are assessed using self-report scales) and developmental trajectories. Academic level serves as a key moderating variable: high school students benefit more from STEM interventions, consistent with social cognitive theory and Cognitive Load Theory, which emphasise the importance of autonomy and competence needs at this developmental stage. The overall effect size (d = 0.46) can serve as a broad contextual reference but is constrained by the aggregation of multiple constructs. From a practical perspective, these findings support tailoring STEM curricula to target outcomes (e.g., prioritising problem-based approaches to cultivate cognitive skills in high school students) and optimising assessment tools to capture domain-specific effects. From a theoretical perspective, they underscore the importance of decomposing learning outcomes to avoid obscuring meaningful differences in STEM impacts. Future research should explore residual heterogeneity in subgroup analyses (e.g., teacher training, cultural factors) and validate intervention models targeting specific outcomes to advance evidence-based STEM education development. Revised conclusion: This exploratory meta-analysis advances understanding of STEM education’s effects by identifying key patterns in heterogeneous literature. Its value lies not in providing definitive effect sizes but in highlighting critical moderators (outcome type and academic level) and residual gaps (unmeasured contextual factors) that demand further investigation.

There are also some limitations in this study. Only journal papers from three databases were selected for this study. In the future, the study should not only include journal papers, but also conference papers and dissertations. More literature in other languages should be absorbed to make comprehensive comparisons. The differences in the effects of different outcome variables should be investigated (self-efficacy, motivation for learning, creativity level, etc.), and the learning effects of STEM education-assisted students should be explored in a multifaceted perspective. The significant heterogeneity among the studies suggests that the effect size may be highly context-dependent, and future research should employ meta-regression and other methods to further explore this heterogeneity. The theoretical framework of this study also lacks sufficient explanation of the interactive mechanisms between the emotional and cognitive domains (e.g., how self-efficacy mediates the effects of STEM interventions). The high heterogeneity of the original studies raised concerns about the conceptual consistency of the meta-analysis. Although subgroup analysis reduced this heterogeneity, unmeasured variables remained, simplifying the complex and context-dependent effects of the statistical meta-analysis. Therefore, narrative synthesis is essential for interpreting these findings. The impact of STEM education is context-specific, and future research should focus on context-specific mechanisms rather than the magnitude of universal effects. Additionally, there is a lack of theoretical explanation for the high effectiveness of short-term interventions, which may require the introduction of immersive learning theory (Chen et al., 2016) to further elucidate the impact of high-intensity short-term interventions on neuroplasticity. In addition, some missing data when combing through the literature prevented this study from exploring some specific moderating variables (gender, creativity, ability classification, etc.). In the future, when conditions are sufficient, the impact of STEM education on student learning outcomes can be comprehensively analysed. In addition, a key limitation lies in the aggregation of different outcome constructs. Cognitive, non-cognitive, and skill performance differ in terms of measurement precision (e.g., objective tests versus self-reports) and sensitivity to STEM interventions, which may lead to biases in the overall effect size. Future research should prioritise separate analyses for each outcome type to avoid confounding their unique mechanisms. A key limitation is the residual heterogeneity in subgroup analyses, which affects the interpretability of findings. Even after stratifying by outcome type and academic level, heterogeneity remains substantial. This indicates unmeasured variables—such as teacher expertise in STEM integration, school resource availability, or cultural norms around hands-on learning—that may influence effect sizes. These unobserved factors limit our ability to draw clear conclusions about the varying effectiveness of STEM education in different contexts, as the remaining variation cannot be fully explained by measured moderators. Given these challenges, the study is best characterized as exploratory rather than confirmatory. It identifies meaningful patterns (e.g., stronger effects on high school cognitive ability) but does not confirm causal mechanisms, as residual heterogeneity suggests complex, context-dependent relationships that require more controlled designs to disentangle. Additionally, the aggregation of diverse constructs (cognitive, non-cognitive, skill performance) in the overall effect size introduces interpretive ambiguity, as these outcomes have distinct measurement properties and theoretical foundations. While subgroup analyses mitigate this issue, they do not eliminate it, reinforcing the need for future studies to focus on single outcome types to enhance precision.

## Data Availability

The original contributions presented in the study are included in the article/Supplementary material, further inquiries can be directed to the corresponding author.

## References

[ref1] Abalde M. A. (2014). School size policies: a literature review.

[ref3] Aldilla E. Asrizal A. Usmeldi U. (2023). Meta-analysis of the STEM application effect on students' creative thinking. Indones. J. Sci. Math. Educ. 6, 165–176. doi: 10.24042/ijsme.v6i2.16218

[ref5] Bandura A. (1986). Social foundations of thought and action. Englewood Cliffs, NJ: Prentice Hall, 2.

[ref6] Bandura A. (2006). “Guide for constructing self-efficacy scales” in Self-efficacy beliefs of adolescents, vol. 5, 307–337.

[ref7] Bloom B. S. Engelhart M. D. Furst E. Hill W. H. Krathwohl D. R. (1956). Handbook I: Cognitive domain. New York: David McKay, 483–498.

[ref8] Borenstein M. Hedges L. V. Higgins J. P. Rothstein H. R. (2021). Introduction to meta-analysis: John Wiley & Sons.

[ref9] Bowman S. Mckinstry C. Howie L. Mcgorry P. (2020). Expanding the search for emerging mental ill health to safeguard student potential and vocational success in high school: a narrative review. Early Interv. Psychiatry 14, 655–676. doi: 10.1111/eip.12928, PMID: 32026624

[ref10] Bransford J. D. Vye N. Kinzer C. Risko V. (2013). “Teaching thinking and content knowledge: toward an integrated approach” in Dimensions of thinking and cognitive instruction (Routledge), 381–413.

[ref11] Capraro R. M. Capraro M. M. Scheurich J. J. Jones M. Morgan J. Huggins K. S. . (2016). Impact of sustained professional development in STEM on outcome measures in a diverse urban district. J. Educ. Res. 109, 181–196. doi: 10.1080/00220671.2014.936997

[ref12] Cervetti G. N. Barber J. Dorph R. Pearson P. D. Goldschmidt P. G. (2012). The impact of an integrated approach to science and literacy in elementary school classrooms. J. Res. Sci. Teach. 49, 631–658. doi: 10.1002/tea.21015

[ref13] Chen J. A. Tutwiler M. S. Metcalf S. J. Kamarainen A. Grotzer T. Dede C. (2016). A multi-user virtual environment to support students' self-efficacy and interest in science: a latent growth model analysis. Learn. Instr. 41, 11–22. doi: 10.1016/j.learninstruc.2015.09.007

[ref14] Chingos M. M. (2013). Class size and student outcomes: research and policy implications. J. Policy Anal. Manage. 32, 411–438. doi: 10.1002/pam.21677

[ref15] Coban M. Bolat Y. I. Goksu I. (2022). The potential of immersive virtual reality to enhance learning: a meta-analysis. Educ. Res. Rev. 36:100452. doi: 10.1016/j.edurev.2022.100452

[ref16] Cohen J. (1988). Statistical power analysis for the behavioral sciences. Second Edn. Hillsdale, NJ: La Wrence Erlabaum Associates, Publishers.

[ref17] Cohen J. (1992a). A power primer. Psychol. Bull. 112, 155–159. doi: 10.1037/0033-2909.112.1.155, PMID: 19565683

[ref18] Cohen J. (1992b). Statistical power analysis. Curr. Dir. Psychol. Sci. 1, 98–101. doi: 10.1111/1467-8721.ep10768783

[ref19] Considine S. L. (2014). *Utilizing* STEM *experiential learning to influence attitudes, skills, and knowledge in urban high school*: Capella University.

[ref20] Cooper H. Hedges L. V. Valentine J. C. (2019). The handbook of research synthesis and meta-analysis: Russell Sage Foundation.

[ref21] Copas J. Shi J. Q. (2000). Meta-analysis, funnel plots and sensitivity analysis. Biostatistics 1, 247–262. doi: 10.1093/biostatistics/1.3.247, PMID: 12933507

[ref22] D’angelo C. Rutstein D. Harris C. Bernard R. Borokhovski E. Haertel G. (2014). Simulations for STEM learning: systematic review and meta-analysis. Menlo Park, CA: Sri International, 1–5.

[ref23] Diamond A. (2013). Executive functions. Annu. Rev. Psychol. 64, 135–168. doi: 10.1146/annurev-psych-113011-143750, PMID: 23020641 PMC4084861

[ref24] Du Y. (2020). Study on cultivating college students' English autonomous learning ability under the flipped classroom model. Engl. Lang. Teach. 13, 13–19. doi: 10.5539/elt.v13n6p13

[ref25] Garzón J. Acevedo J. (2019). Meta-analysis of the impact of augmented reality on students’ learning gains. Educ. Res. Rev. 27, 244–260. doi: 10.1016/j.edurev.2019.04.001

[ref26] Gülen S. (2019). The effect of STEM roles on the solution of daily life problems. Particip. Educ. Res. 6, 37–50.

[ref27] Huitt W. Hummel J. (2003). Piaget's theory of cognitive development. Educ. Psychol. Interact. 3, 1–5. doi: 10.1007/978-0-387-79061-9_2164

[ref28] Jonassen D. (2011). Supporting problem solving in PBL. Interdiscip. J. Probl.-based Learn. 5, 95–119. doi: 10.7771/1541-5015.1256

[ref30] Khalil R. Y. Tairab H. Qablan A. Alarabi K. Mansour Y. (2023). STEM-based curriculum and creative thinking in high school students. Educ. Sci. 13:1195. doi: 10.3390/educsci13121195

[ref31] Kong S. C. (2014). Developing information literacy and critical thinking skills through domain knowledge learning in digital classrooms: an experience of practicing flipped classroom strategy. Comput. Educ. 78, 160–173. doi: 10.1016/j.compedu.2014.05.009

[ref32] Kurt M. Benzer S. (2020). An investigation on the effect of STEM practices on sixth grade students' academic achievement, problem solving skills, and attitudes towards STEM. J. Sci. Learn. 3, 79–88. doi: 10.17509/jsl.v3i2.21419

[ref33] Lim S. F. W. Jin X. Srai J. S. (2018). Consumer-driven e-commerce: a literature review, design framework, and research agenda on last-mile logistics models. Int. J. Phys. Distrib. Logist. Manage. 48, 308–332. doi: 10.1108/IJPDLM-02-2017-0081

[ref34] Lin Y.-T. Wang M.-T. Wu C.-C. (2019). Design and implementation of interdisciplinary STEM instruction: teaching programming by computational physics. Asia Pac. Educ. Res. 28, 77–91. doi: 10.1007/s40299-018-0415-0

[ref35] Linnenbrink-Garcia L. Durik A. M. Conley A. M. Barron K. E. Tauer J. M. Karabenick S. A. . (2010). Measuring situational interest in academic domains. Educ. Psychol. Meas. 70, 647–671. doi: 10.1177/0013164409355699

[ref37] Lynch K. Hill H. C. Gonzalez K. E. Pollard C. (2019). Strengthening the research base that informs STEM instructional improvement efforts: a meta-analysis. Educ. Eval. Policy Anal. 41, 260–293. doi: 10.3102/0162373719849044

[ref38] Maass K. Geiger V. Ariza M. R. Goos M. (2019). The role of mathematics in interdisciplinary STEM education. ZDM 51, 869–884. doi: 10.1007/s11858-019-01100-5

[ref39] Micari M. Pazos P. (2021). Beyond grades: improving college students’ social-cognitive outcomes in STEM through a collaborative learning environment. Learn. Environ. Res. 24, 123–136. doi: 10.1007/s10984-020-09325-y

[ref41] Mustafa N. Ismail Z. Tasir Z. Mohamad Said M. N. H. (2016). A meta-analysis on effective strategies for integrated STEM education. Adv. Sci. Lett. 22, 4225–4228. doi: 10.1166/asl.2016.8111

[ref9001] National Research Council (2014). Division on Earth, Life Studies, Board on Life Sciences, & Committee on Key Challenge Areas for Convergence. Convergence: Facilitating transdisciplinary integration of life sciences, physical sciences, engineering, and beyond. 24830066

[ref42] Noor U. Younas M. Saleh Aldayel H. Menhas R. Qingyu X. (2022). Learning behavior, digital platforms for learning and its impact on university student’s motivations and knowledge development. Front. Psychol. 13:933974. doi: 10.3389/fpsyg.2022.933974, PMID: 36506979 PMC9726725

[ref43] Oprean D. Balakrishnan B. (2020). From engagement to user experience: A theoretical perspective towards immersive learning.

[ref45] Ortiz-Revilla J. Greca I. M. Arriassecq I. (2022). A theoretical framework for integrated STEM education. Sci. Educ. 31, 383–404. doi: 10.1007/s11191-021-00242-x

[ref46] Pellas N. Kazanidis I. Konstantinou N. Georgiou G. (2017). Exploring the educational potential of three-dimensional multi-user virtual worlds for STEM education: a mixed-method systematic literature review. Educ. Inf. Technol. 22, 2235–2279. doi: 10.1007/s10639-016-9537-2

[ref47] Pelletier J. P. Jovanovic D. Fernandes J. C. Manning P. Connor J. R. Currie M. G. . (1998). Reduced progression of experimental osteoarthritis in vivo by selective inhibition of inducible nitric oxide synthase. Arthritis Rheum. 41, 1275–1286. doi: 10.1002/1529-0131(199807)41:7<1275::AID-ART19>3.0.CO;2-T 9663486

[ref48] Petitti D. B. (2001). Approaches to heterogeneity in meta-analysis. Stat. Med. 20, 3625–3633. doi: 10.1002/sim.1091, PMID: 11746342

[ref49] Piaget J. Duckworth E. (1970). Genetic epistemology. Am. Behav. Sci. 13, 459–480. doi: 10.1177/000276427001300320

[ref50] Pigott T. D. Polanin J. R. (2020). Methodological guidance paper: high-quality meta-analysis in a systematic review. Rev. Educ. Res. 90, 24–46. doi: 10.3102/0034654319877153

[ref51] Qiu X. B. Shan C. Yao J. Fu Q. K. (2024). The effects of virtual reality on EFL learning: a meta-analysis. Educ. Inf. Technol. 29, 1379–1405. doi: 10.1007/s10639-023-11738-0

[ref52] Robinson L. (2020). The STEM selfing process: nondigital and digital determinants of aspirational STEM futures. Am. Behav. Sci. 64, 950–972. doi: 10.1177/0002764220919150

[ref53] Rosenthal R. (1979). The file drawer problem and tolerance for null results. Psychol. Bull. 86, 638–641. doi: 10.1037/0033-2909.86.3.638

[ref54] Rosenthal R. Dimatteo M. R. (2001). Meta-analysis: recent developments in quantitative methods for literature reviews. Annu. Rev. Psychol. 52, 59–82. doi: 10.1146/annurev.psych.52.1.59, PMID: 11148299

[ref55] Rücker G. Schwarzer G. Carpenter J. R. Schumacher M. (2008). Undue reliance on I 2 in assessing heterogeneity may mislead. BMC Med. Res. Methodol. 8, 1–9. doi: 10.1186/1471-2288-8-79 19036172 PMC2648991

[ref56] Ruengtam P. (2018). Cooperative/collaborative learning technique in theoretical subjects of interior architecture program. J. Asian Behav. Stud. 3, 27–39. doi: 10.21834/jabs.v3i7.255

[ref57] Runco M. A. Pritzker S. R. (2020). Encyclopedia of creativity: Academic Press.

[ref58] Ryan R. M. Deci E. L. (2000). Self-determination theory and the facilitation of intrinsic motivation, social development, and well-being. Am. Psychol. 55, 68–78. doi: 10.1037/0003-066X.55.1.68, PMID: 11392867

[ref60] Scheerens J. (2016). Educational effectiveness and ineffectiveness. A critical review of the knowledge base, 389.

[ref61] Schwarzer R. Jerusalem M. (1995). “Generalized self-efficacy scale” in Measures in health psychology: a user’s portfolio. Causal and control beliefs. eds. Weinman J. Wright S. Johnston M., 35–37.

[ref62] Sgro C. M. Bobowski T. Oliveira A. W. (2020). “Current praxis and conceptualization of STEM education: a call for greater clarity in integrated curriculum development” in Critical questions in STEM education, 185–210.

[ref63] Sirajudin N. Suratno J. (2021). Developing creativity through STEM education. J. Phys. Conf. Ser. 1806:012211. doi: 10.1088/1742-6596/1806/1/012211, PMID: 40712154

[ref64] Suárez-Eiroa B. Fernández E. Méndez-Martínez G. Soto-Oñate D. (2019). Operational principles of circular economy for sustainable development: linking theory and practice. J. Clean. Prod. 214, 952–961. doi: 10.1016/j.jclepro.2018.12.271

[ref65] Sweller J. (1994). Cognitive load theory, learning difficulty, and instructional design. Learn. Instr. 4, 295–312. doi: 10.1016/0959-4752(94)90003-5

[ref67] Thornton A. Lee P. (2000). Publication bias in meta-analysis: its causes and consequences. J. Clin. Epidemiol. 53, 207–216. doi: 10.1016/S0895-4356(99)00161-4, PMID: 10729693

[ref68] Townes T. C. (2016). The consequences of creativity in the classroom: The impact of arts integration on student learning: Union University.

[ref69] Wahono B. Lin P.-L. Chang C.-Y. (2020). Evidence of STEM enactment effectiveness in Asian student learning outcomes. *Int. J.* STEM *Educ.* 7:36. doi: 10.1186/s40594-020-00236-1

[ref70] Wohlin C. (2014). Guidelines for snowballing in systematic literature studies and a replication in software engineering. Proceedings of the 18th international conference on evaluation and assessment in software engineering, pp. 1–10.

[ref71] Wu B. Yu X. Gu X. (2020). Effectiveness of immersive virtual reality using head-mounted displays on learning performance: a meta-analysis. Br. J. Educ. Technol. 51, 1991–2005. doi: 10.1111/bjet.13023

[ref75] Yu Z. (2023). A meta-analysis of the effect of virtual reality technology use in education. Interact. Learn. Environ. 31, 4956–4976. doi: 10.1080/10494820.2021.1989466

[ref73] Younas M. Dong Y. Zhao G. Menhas R. Luan L. Noor U. (2025). Unveiling digital transformation and teaching prowess in English education during Covid-19 with structural equation modelling. Eur. J. Educ. 60:e12818. doi: 10.1111/ejed.12818

[ref74] Younas M. Noor U. Zhou X. Menhas R. Qingyu X. (2022). Covid-19, students satisfaction about e-learning and academic achievement: mediating analysis of online influencing factors. Front. Psychol. 13:948061. doi: 10.3389/fpsyg.2022.948061, PMID: 36081717 PMC9444837

[ref9002] Yildirim B. Sidekli S. (2018). STEM applications in mathematics education: The effect of STEM applications on different dependent variables.

[ref76] Zhang H. Yu L. Ji M. Cui Y. Liu D. Li Y. . (2020). Investigating high school students’ perceptions and presences under Vr learning environment. Interact. Learn. Environ. 28, 635–655. doi: 10.1080/10494820.2019.1709211

[ref77] Zhao M. Lu X. Zhang Q. Zhao R. Wu B. Huang S. . (2024). Effects of exergames on student physical education learning in the context of the artificial intelligence era: a meta-analysis. Sci. Rep. 14:7115. doi: 10.1038/s41598-024-57357-8, PMID: 38531948 PMC10965939

[ref78] Zhou X. Younas M. Omar A. Guan L. (2022). Can second language metaphorical competence be taught through instructional intervention? A meta-analysis. Front. Psychol. 13:1065803. doi: 10.3389/fpsyg.2022.1065803, PMID: 36571037 PMC9769122

