# Supplementary material for: Systematic review and meta-analysis of the impact of STEM education on students learning outcomes
Source: Front Psychol. 2025 Aug 13;16:1579474. doi: 10.3389/fpsyg.2025.1579474 (PMC12381834; doi:10.3389/fpsyg.2025.1579474)
Supplement: Supplementary file 1 [file Supplementary_file_1.docx]

Supplementary Material

# Supplementary Data

| **Author (Year)** | **Sample size** | **Academic level** | **Subjects** | **Experimental period** | **Teaching method** | **Experimental results** |
| --- | --- | --- | --- | --- | --- | --- |
| Asrizal et al. (2023) | E20, C20 | Primary Schools | Technology | <1 weeks | Project-orientated | Positive impact |
| Ha et al. (2023) | E31, C36 | High Schools | Engineering | <1 weeks | Problem-orientated | Positive impact |
| Khalil et al. (2023) | E48, C46 | High Schools | Technology | 1–5 weeks | Inquiry -orientated | Positive impact |
| Abdurrahman et al. (2023) | E31, C36 | High Schools | Engineering | <1 weeks | Problem-orientated | Positive impact |
| Awad (2023) | E120, C120 | High Schools | Science | >10 weeks | Project-orientated | Positive impact |
| Chang and Chen (2022) | E42, C42 | High Schools | Science | 1–5 weeks | Project-orientated | Positive impact |
| Minarti et al. (2022) | E36, C36 | Primary Schools | Technology | <1 weeks | Project-orientated | No significant difference |
| Sirajudin and Suratno (2021) | E12, C12 | Universities | Science | <1 weeks | Project-orientated | Positive impact |
| Micari and Pazos (2021) | E604, C676 | Universities | Technology | 5–10 weeks | Problem-orientated | Positive impact |
| Kencana and Syukri (2020) | E101, C102 | High Schools | Technology | >10 weeks | Inquiry -orientated | Positive impact |
| Kurt and Benzer (2020) | E13, C13 | Primary Schools | Science | 5–10 weeks | Problem-orientated | Positive impact |
| Oren et al. (2020) | E9, C27 | High Schools | Science | >10 weeks | Project-orientated | No significant difference |
| Oren et al. (2020) | E18, C52 | High Schools | Mathematics | >10 weeks | Project-orientated | Positive impact |
| Lin et al. (2019) | E78, C71 | High Schools | Engineering | >10 weeks | Project-orientated | Positive impact |
| Özcan and Koca (2019) | E20, C13 | High Schools | Science | >10 weeks | Project-orientated | Positive impact |
| Yaki et al. (2019) | E51, C49 | High Schools | Science | 5–10 weeks | Project-orientated | No significant difference |
| Gülen and Yaman (2019) | E20, C20 | High Schools | Science | >10 weeks | Project-orientated | Positive impact |
| Gülen (2019) | E20, C18 | High Schools | Engineering | 1–5 weeks | Project-orientated | No significant difference |
| Gülhan and Şahin (2018) | E30, C33 | High Schools | Science | 1–5 weeks | Project-orientated | Positive impact |
| Proudfoot et al. (2018) | E17, C428 | Primary Schools | Mathematics | >10 weeks | Problem-orientated | No significant difference |
| Ojaleye and Awofala (2018) | E96, C116 | High Schools | Mathematics | >10 weeks | Problem-orientated | Positive impact |
| Yıldırım and Sidekli (2018) | E29, C29 | Universities | Mathematics | >10 weeks | Project-orientated | Positive impact |
| Sarican and Akgunduz (2018) | E22, C22 | Primary Schools | Science | >10 weeks | Project-orientated | No significant difference |
| Toma and Greca (2018) | E55, C41 | Primary Schools | Engineering | >10 weeks | Project-orientated | Positive impact |
| Acara et al. (2018) | E25, C25 | Primary Schools | Mathematics | 5–10 weeks | Inquiry -orientated | Positive impact |
| Yildirim and Selvi (2017) | E26, C22 | High Schools | Science | 5–10 weeks | Inquiry -orientated | Positive impact |
| Lou et al. (2017) | E60, C60 | High Schools | Science | 5–10 weeks | Project-orientated | Positive impact |
| Arsad et al. (2017) | E56, C58 | Primary Schools | Engineering | 1–5 weeks | Inquiry -orientated | No significant difference |
| Fan and Yu (2017) | E171, C161 | High Schools | Engineering | >10 weeks | Project-orientated | Positive impact |
| Tati et al. (2017) | E36, C36 | High Schools | Science | <1 weeks | Project-orientated | Positive impact |
| Sunyoung et al. (2016) | E661, C526 | Universities | Mathematics | >10 weeks | Project-orientated | Positive impact |
| Rasul et al. (2016) | E125, C125 | High Schools | Technology | <1 weeks | Project-orientated | Positive impact |
| ONER et al. (2016) | E1481, C1481 | High Schools | Mathematics | >10 weeks | Project-orientated | No significant difference |
| Corlu and Aydin (2016) | E125, C125 | Universities | Mathematics | >10 weeks | Problem-orientated | Positive impact |
| Açışlı (2016) | E20, C20 | High Schools | Science | <1 weeks | Inquiry -orientated | Positive impact |
| Tolliver (2016) | E64, C53 | Primary Schools | Mathematics | 1–5 weeks | Problem-orientated | No significant difference |
| Townes (2016) | E27, C27 | High Schools | Technology | >10 weeks | Project-orientated | No significant difference |
| Wade-Shepherd (2016) | E916, C916 | High Schools | Mathematics | >10 weeks | Project-orientated | Positive impact |
| Robinson (2016) | E54, C54 | High Schools | Technology | 1–5 weeks | Problem-orientated | Positive impact |
| Yildirim and Selvi (2016) | E76, C76 | Primary Schools | Technology | >10 weeks | Project-orientated | Positive impact |
| Erdoğan and Stuessy (2015) | E9004, C19155 | High Schools | Mathematics | >10 weeks | Inquiry -orientated | No significant difference |
| Erdoğan and Stuessy (2015) | E9004, C19155 | High Schools | Science | >10 weeks | Inquiry -orientated | No significant difference |
| Harris et al. (2015) | E46, C26 | Primary Schools | Science | >10 weeks | Project-orientated | No significant difference |
| Maxwell et al. (2015) | E22, C20 | Primary Schools | Science | 5–10 weeks | Inquiry -orientated | Positive impact |
| Bicer et al. (2015) | E1506, C1520 | High Schools | Mathematics | >10 weeks | Problem-orientated | Positive impact |
| Parker et al. (2015) | E35, C24 | Universities | Science | >10 weeks | Inquiry -orientated | Positive impact |
| Karahan et al. (2015) | E21, C21 | High Schools | Science | >10 weeks | Project-orientated | Positive impact |
| Abdullah et al. (2014) | E96, C97 | Primary Schools | Mathematics | >10 weeks | Problem-orientated | Positive impact |
| Robinson et al. (2014) | E38, C38 | Primary Schools | Science | >10 weeks | Project-orientated | Positive impact |
| Judson (2014) | E53, C3681 | High Schools | Mathematics | >10 weeks | Problem-orientated | Positive impact |
| Kong and Huo (2014) | E25, C25 | Primary Schools | Technology | 1–5 weeks | Project-orientated | Positive impact |
| Cotabish et al. (2013) | E42, C54 | Primary Schools | Mathematics | >10 weeks | Problem-orientated | Positive impact |
| Cotabish et al. (2013) | E818, C932 | Primary Schools | Science | 1–5 weeks | Inquiry-orientated | Positive impact |
| Cotabish et al. (2013) | E139, C139 | Primary Schools | Science | >10 weeks | Inquiry -orientated | Positive impact |
| Park and Yoo (2013) | E26, C26 | Primary Schools | Technology | 5–10 weeks | Inquiry -orientated | No significant difference |
| Kim et al. (2012) | E88, C106 | Primary Schools | Science | >10 weeks | Inquiry -orientated | Positive impact |
| Cervetti et al. (2012) | E976, C937 | Primary Schools | Technology | 1–5 weeks | Project-orientated | No significant difference |
| Kim and Choi (2012) | E18, C20 | Primary Schools | Science | 5–10 weeks | Inquiry -orientated | No significant difference |
| Ruiz-Primo et al. (2011) | E166, C166 | Universities | Science | >10 weeks | Project-orientated | No significant difference |
| Nugent et al. (2010) | E124, C124 | High Schools | Science | <1 weeks | Inquiry -orientated | No significant difference |
| Riskowski et al. (2009) | E126, C126 | High Schools | Science | 1–5 weeks | Project-orientated | Positive impact |
| Apedoe et al. (2008) | E271, C271 | High Schools | Science | 5–10 weeks | Problem-orientated | Positive impact |
| Lam et al. (2008) | E21, C21 | High Schools | Technology | >10 weeks | Project-orientated | Positive impact |
| Mehalik et al. (2008) | E587, C466 | High Schools | Science | >10 weeks | Problem-orientated | Positive impact |
| Sullivan (2008) | E26, C26 | High Schools | Science | >10 weeks | Project-orientated | Positive impact |
| Cole and Espinoza (2008) | E146, C146 | Universities | Technology | 1–5 weeks | Problem-orientated | Positive impact |

| **Author (Year)** | **E** | **M** | **SD** | **C** | **M** | **SD** |
| --- | --- | --- | --- | --- | --- | --- |
| Asrizal et al. (2023) | 20 | 82.60 | 10.53 | 20 | 73.80 | 13.13 |
| Ha et al. (2023) | 31 | 82.42 | 5.60 | 36 | 75.28 | 7.74 |
| Khalil et al. (2023) | 48 | 1.20 | 1.00 | 46 | 0.86 | 1.02 |
| Abdurrahman et al. (2023) | 31 | 82.42 | 5.60 | 36 | 75.28 | 7.74 |
| Awad (2023) | 120 | 81.96 | 12.70 | 120 | 66.40 | 19.20 |
| Chang and Chen (2022) | 42 | 4.03 | 1.03 | 42 | 3.77 | 1.01 |
| Minarti et al. (2022) | 36 | 11.08 | 2.45 | 36 | 11.00 | 1.83 |
| Sirajudin and Suratno (2021) | 12 | 22.50 | 1.73 | 12 | 17.92 | 4.98 |
| Micari and Pazos (2021) | 604 | 4.00 | 1.11 | 676 | 3.78 | 1.14 |
| Kencana and Syukri (2020) | 101 | 79.90 | 11.31 | 102 | 63.00 | 10.06 |
| Kurt and Benzer (2020) | 13 | 3.74 | 0.56 | 13 | 2.76 | 0.55 |
| Oren et al. (2020) | 9 | 0.94 | 0.08 | 27 | 0.93 | 0.06 |
| Oren et al. (2020) | 18 | 0.94 | 0.06 | 52 | 0.91 | 0.11 |
| Lin et al. (2019) | 78 | 76.62 | 17.61 | 71 | 69.83 | 17.66 |
| Özcan and Koca (2019) | 20 | 148.60 | 12.75 | 13 | 142.30 | 24.61 |
| Yaki et al. (2019) | 51 | 40.35 | 6.89 | 49 | 39.49 | 6.32 |
| Gülen and Yaman (2019) | 20 | 24.50 | 4.90 | 20 | 16.50 | 3.30 |
| GÜLEN (2019) | 20 | 21.35 | 1.3 | 18 | 17.44 | 1.2 |
| Gülhan and Şahin (2018) | 30 | 14.53 | 4.96 | 33 | 11.06 | 4.35 |
| Proudfoot et al. (2018) | 17 | 47.50 | 1.84 | 428 | 44.00 | 2.87 |
| Ojaleye and Awofala (2018) | 96 | 24.25 | 9.34 | 116 | 17.47 | 5.10 |
| Yıldırım and Sidekli (2018) | 29 | 16.93 | 2.75 | 29 | 17.03 | 2.64 |
| Sarican and Akgunduz (2018) | 22 | 11.00 | 2.94 | 22 | 10.45 | 1.81 |
| Toma and Greca (2018) | 55 | 10 | 3.69 | 41 | 9.9 | 3.63 |
| Acara et al. (2018) | 25 | 15.92 | 2.85 | 25 | 13.36 | 2.01 |
| Yildirim and Selvi (2017) | 26 | 7.57 | 3.47 | 22 | 6.64 | 1.76 |
| Lou et al. (2017) | 60 | 28.28 | 5.15 | 60 | 26.68 | 4.47 |
| Arsad et al. (2017) | 56 | 4.19 | 1.03 | 58 | 4.20 | 0.72 |
| Fan and Yu (2017) | 171 | 55.35 | 15.22 | 161 | 42.27 | 9.07 |
| Tati et al. (2017) | 36 | 1.67 | 0.56 | 36 | 1.19 | 0.57 |
| Sunyoung et al. (2016) | 661 | 34.84 | 10.50 | 526 | 32.56 | 10.80 |
| Rasul et al. (2016) | 125 | 4.14 | 0.45 | 125 | 4.05 | 0.33 |
| ONER et al. (2016) | 1481 | 2365.31 | 172.02 | 1481 | 2361.48 | 236.20 |
| Corlu and Aydin (2016) | 125 | 12.49 | 2.69 | 125 | 10.17 | 2.30 |
| Açışlı (2016) | 20 | 17.25 | 2.26 | 20 | 13.20 | 2.44 |
| Tolliver (2016) | 64 | 60.57 | 16.13 | 53 | 57.70 | 10.97 |
| Townes (2016) | 27 | 25.67 | 9.43 | 27 | 23.46 | 6.65 |
| Wade-Shepherd (2016) | 916 | 62.77 | 17.37 | 916 | 57.65 | 19.83 |
| Robinson (2016) | 54 | 3926 | 1358 | 54 | 3204 | 1294 |
| Yildirim and Selvi (2016) | 76 | 51.4 | 10.75 | 76 | 43.37 | 14.40 |
| Erdoğan and Stuessy (2015) | 9004 | 2253 | 246 | 19155 | 2228 | 236 |
| Erdoğan and Stuessy (2015) | 9004 | 2249 | 208 | 19155 | 2239 | 204 |
| Harris et al. (2015) | 46 | 77.42 | 12.93 | 26 | 79.17 | 13.63 |
| Maxwell et al. (2015) | 22 | 78.82 | 15.73 | 20 | 51.14 | 14.02 |
| Bicer et al. (2015) | 1506 | 10.27 | 1.64 | 1520 | 9.65 | 1.27 |
| Parker et al. (2015) | 35 | 10.50 | 2.5 | 24 | 9.33 | 2.34 |
| Karahan et al. (2015) | 21 | 30.90 | 2.14 | 21 | 27.09 | 5.84 |
| Abdullah et al. (2014) | 96 | 75.75 | 11.45 | 97 | 60.32 | 16.02 |
| Robinson et al. (2014) | 38 | 9.11 | 1.94 | 38 | 8.53 | 1.61 |
| Judson (2014) | 53 | 5.98 | 2.10 | 3681 | 5.53 | 1.90 |
| Kong and Huo (2014) | 25 | 23.25 | 6.53 | 25 | 19.40 | 5.48 |
| Cotabish et al. (2013) | 42 | 43.79 | 14.46 | 54 | 34.96 | 9.62 |
| Cotabish et al. (2013) | 818 | 6.44 | 3.18 | 932 | 5.33 | 3.07 |
| Cotabish et al. (2013) | 139 | 9.27 | 3.55 | 139 | 8.45 | 3.40 |
| Park and Yoo (2013) | 26 | 13.73 | 1.88 | 26 | 13.69 | 1.33 |
| Kim et al. (2012) | 88 | 16.70 | 5.60 | 106 | 14.30 | 6.10 |
| Cervetti et al. (2012) | 976 | 15.41 | 3.45 | 937 | 14.05 | 2.58 |
| Kim and Choi (2012) | 18 | 23.54 | 3.45 | 20 | 43.56 | 4.57 |
| Ruiz-Primo et al. (2011) | 166 | 0.47 | 0.54 | 166 | 0.43 | 0.49 |
| Nugent et al. (2010) | 124 | 4.23 | 0.53 | 124 | 4.12 | 0.46 |
| Riskowski et al. (2009) | 126 | 4.32 | 0.51 | 126 | 3.94 | 0.47 |
| Apedoe et al. (2008) | 271 | 27.65 | 4.25 | 271 | 25.56 | 3.08 |
| Lam et al. (2008) | 21 | 9.71 | 1.57 | 21 | 8.39 | 1.46 |
| Mehalik et al. (2008) | 587 | 21.39 | 3.16 | 466 | 20.63 | 2.97 |
| Sullivan (2008) | 26 | 25.82 | 4.04 | 26 | 23.09 | 4.14 |
| Cole and Espinoza (2008) | 146 | 11.46 | 2.38 | 146 | 9.76 | 1.75 |

Abdullah, N., Halim, L., & Zakaria, E. (2014). VStops: A thinking strategy and visual representation approach in mathematical word problem solving toward enhancing STEM literacy. *Eurasia Journal of Mathematics, Science and Technology Education*, *10*(3), 165-174.

Abdurrahman, A., Maulina, H., Nurulsari, N., Sukamto, I., Umam, A. N., & Mulyana, K. M. (2023). Impacts of integrating engineering design process into STEM makerspace on renewable energy unit to foster students’ system thinking skills. *Heliyon*, *9*(4).

Acara, D., Tertemizb, N., & Taşdemirc, A. (2018). The effects of STEM training on the academic achievement of 4th graders in science and mathematics and their views on STEM training teachers. *International electronic journal of elementary education*, *10*(4), 505-513.

Açışlı, S. (2016). Investigation of the effect of robotic applications in elementary education. *The Eurasia Proceedings of Educational and Social Sciences*, *4*, 391-394.

Apedoe, X. S., Reynolds, B., Ellefson, M. R., & Schunn, C. D. (2008). Bringing engineering design into high school science classrooms: The heating/cooling unit. *Journal of Science Education and Technology*, *17*, 454-465.

Arsad, N. M., Osman, K., & Embi, M. A. (2017). Effect Of Stem-5e Learning Cycle (Am-Stem Kids Module) In Fostering Noble Values Among Elementary School Children. *The Eurasia Proceedings of Educational and Social Sciences*, *6*, 160-166.

Asrizal, A., Annisa, N., Festiyed, F., Ashel, H., & Amnah, R. (2023). STEM-integrated physics digital teaching material to develop conceptual understanding and new literacy of students. *Eurasia Journal of Mathematics, Science and Technology Education*, *19*(7), em2289.

Awad, N. (2023). Exploring STEM integration: assessing the effectiveness of an interdisciplinary informal program in fostering students’ performance and inspiration. *Research in Science & Technological Education*, *41*(2), 675-699.

Bicer, A., Navruz, B., Capraro, R. M., Capraro, M. M., Oner, T., & Boedeker, P. (2015). STEM schools vs. non-STEM schools: Comparing students' mathematics growth rate on high-stakes test performance. *International Journal of New Trends in Education and Their Implications*, *6*(1), 138-150.

Cervetti, G. N., Barber, J., Dorph, R., Pearson, P. D., & Goldschmidt, P. G. (2012). The impact of an integrated approach to science and literacy in elementary school classrooms. *Journal of research in science teaching*, *49*(5), 631-658.

Chang, C.-C., & Chen, Y. (2022). Using mastery learning theory to develop task-centered hands-on STEM learning of Arduino-based educational robotics: Psychomotor performance and perception by a convergent parallel mixed method. *Interactive Learning Environments*, *30*(9), 1677-1692.

Cole, D., & Espinoza, A. (2008). Examining the academic success of Latino students in science technology engineering and mathematics (STEM) majors. *Journal of College Student Development*, *49*(4), 285-300.

Corlu, M. A., & Aydin, E. (2016). Evaluation of learning gains through integrated STEM projects. *International Journal of Education in Mathematics, Science and Technology*, *4*(1), 20-29.

Cotabish, A., Dailey, D., Robinson, A., & Hughes, G. (2013). The effects of a STEM intervention on elementary students' science knowledge and skills. *School Science and Mathematics*, *113*(5), 215-226.

Erdoğan, N., & Stuessy, C. (2015). Examining the role of ınclusive stem schools in the college and career readiness of students in the united states: a multi-group analysis on the outcome of student achievement.

Fan, S.-C., & Yu, K.-C. (2017). How an integrative STEM curriculum can benefit students in engineering design practices. *International Journal of Technology and Design Education*, *27*, 107-129.

GÜLEN, S. (2019). The effect of STEM roles on the solution of daily life problems. *Participatory Educational Research*, *6*(2), 37-50.

Gülen, S., & Yaman, S. (2019). The effect of integration of STEM disciplines into Toulmin's argumentation model on students’ academic achievement, reflective thinking, and psychomotor skills. *Journal of Turkish Science Education*, *16*(2), 216-230.

Gülhan, F., & Şahin, F. (2018). The effects of STEAM (STEM+ Art) activities 7th grade students’ academic achievement, STEAM attitude and scientific creativities STEAM (STEM+ Sanat) etkinliklerinin 7. sınıf öğrencilerinin akademik başarı, STEAM tutum ve bilimsel yaratıcılıklarına etkisi. *Journal of human sciences*, *15*(3), 1675-1699.

Ha, V. T., Chung, L. H., Hanh, N. V., & Hai, B. M. (2023). Teaching science using argumentation-supported 5e-STEM, 5e-STEM, and conventional didactic methods: Differences in the learning outcomes of middle school students. *Education Sciences*, *13*(3), 247.

Harris, C. J., Penuel, W. R., D'Angelo, C. M., DeBarger, A. H., Gallagher, L. P., Kennedy, C. A., . . . Krajcik, J. S. (2015). Impact of project‐based curriculum materials on student learning in science: Results of a randomized controlled trial. *Journal of research in science teaching*, *52*(10), 1362-1385.

Judson, E. (2014). Effects of transferring to STEM-focused charter and magnet schools on student achievement. *The Journal of Educational Research*, *107*(4), 255-266.

Karahan, E., Bilici, S. C., & Ayçin, Ü. (2015). Integration of media design processes in science, technology, engineering, and mathematics (STEM) education. *Eurasian Journal of Educational Research*, *15*(60), 221-240.

Kencana, M., & Syukri, M. (2020). The effect of science, technology, engineering, and mathematics (STEM) on students’ creative thinking skills. Journal of Physics: Conference Series,

Khalil, R. Y., Tairab, H., Qablan, A., Alarabi, K., & Mansour, Y. (2023). STEM-Based Curriculum and Creative Thinking in High School Students. *Education Sciences*, *13*(12), 1195.

Kim, G.-S., & Choi, S. Y. (2012). The effects of the creative problem solving ability and scientific attitude through the science-based STEAM program in the elementary gifted students. *Journal of Korean elementary science education*, *31*(2), 216-226.

Kim, K. H., VanTassel-Baska, J., Bracken, B. A., Feng, A., Stambaugh, T., & Bland, L. (2012). Project Clarion: Three years of science instruction in Title I schools among K-third grade students. *Research in Science Education*, *42*, 813-829.

Kong, Y. T., & Huo, S.-C. (2014). An effect of STEAM activity programs on science learning interest. *Advanced Science and Technology Letters*, *59*, 41-45.

Kurt, M., & Benzer, S. (2020). An Investigation on the Effect of STEM Practices on Sixth Grade Students' Academic Achievement, Problem Solving Skills, and Attitudes towards STEM. *Journal of Science Learning*, *3*(2), 79-88.

Lam, P., Doverspike, D., Zhao, J., Zhe, J., & Menzemer, C. (2008). An evaluation of a STEM program for middle school students on learning disability related IEPs. *Journal of STEM education*, *9*(1).

Lin, Y.-T., Wang, M.-T., & Wu, C.-C. (2019). Design and implementation of interdisciplinary STEM instruction: Teaching programming by computational physics. *The Asia-Pacific Education Researcher*, *28*, 77-91.

Lou, S.-J., Chou, Y.-C., Shih, R.-C., & Chung, C.-C. (2017). A study of creativity in CaC2 steamship-derived STEM project-based learning. *Eurasia Journal of Mathematics, Science and Technology Education*, *13*(6), 2387-2404.

Maxwell, D. O., Lambeth, D. T., & Cox, J. (2015). Effects of using inquiry-based learning on science achievement for fifth-grade students. Asia-Pacific Forum on Science Learning & Teaching,

Mehalik, M. M., Doppelt, Y., & Schuun, C. D. (2008). Middle‐school science through design‐based learning versus scripted inquiry: Better overall science concept learning and equity gap reduction. *Journal of engineering education*, *97*(1), 71-85.

Micari, M., & Pazos, P. (2021). Beyond grades: improving college students’ social-cognitive outcomes in STEM through a collaborative learning environment. *Learning Environments Research*, *24*(1), 123-136.

Minarti, I. B., Dzakiy, M. A., & Nilautama, D. (2022). The Effect of STEM (Science, Technology, Engineering, and Mathematics) Based Learning Approach on Critical Thinking Skills and Cognitive Learning Outcomes of Class X SMA Negeri 1. *At-Tasyrih: jurnal pendidikan dan hukum Islam*, *8*(2), 126-136.

Nugent, G., Barker, B., Grandgenett, N., & Adamchuk, V. I. (2010). Impact of robotics and geospatial technology interventions on youth STEM learning and attitudes. *Journal of Research on Technology in Education*, *42*(4), 391-408.

Ojaleye, O., & Awofala, A. O. (2018). Blended Learning and Problem-Based Learning Instructional Strategies as Determinants of Senior Secondary School Students' Achievement in Algebra. *International Journal of Research in Education and Science*, *4*(2), 486-501.

ONER, A. T., CAPRARO, R. M., & CAPRARO, M. M. (2016). The effect of T-STEM designation on charter schools: A longitudinal examination of students’ mathematics achievement. *Sakarya University Journal of Education*, *6*(2), 80-96.

Oren, M., Willson, V., Hubert, T., & Capraro, R. M. (2020). Longitudinal analysis of T-STEM academies: How do Texas inclusive STEM academies (T-STEM) perform in mathematics, science, and reading? *International Online Journal of Educational Sciences*, *7*(4).

Özcan, H., & Koca, E. (2019). The impact of teaching the subject “pressure” with STEM approach on the academic achievements of the secondary school 7th grade students and their attitudes towards STEM.

Park, S.-J., & Yoo, P. K. (2013). The effects of the learning motive, interest and science process skills using the'Light'unit on science-based STEAM. *Journal of Korean elementary science education*, *32*(3), 225-238.

Parker, C. E., Stylinski, C. D., Bonney, C. R., Schillaci, R., & McAuliffe, C. (2015). Examining the quality of technology implementation in STEM classrooms: Demonstration of an evaluative framework. *Journal of Research on Technology in Education*, *47*(2), 105-121.

Proudfoot, D. E., Green, M., Otter, J. W., & Cook, D. L. (2018). STEM Certification in Georgia's Schools: A Causal Comparative Study Using the Georgia Student Growth Model. *Georgia Educational Researcher*, *15*(1), 16-39.

Rasul, M. S., Halim, L., & Iksan, Z. (2016). USING STEM INTEGRATED APPROACH TO NURTURE STUDENTS’INTEREST AND 21ST CENTURY SKILLS. *The Eurasia Proceedings of Educational and Social Sciences*, *4*, 313-319.

Riskowski, J. L., Todd, C. D., Wee, B., Dark, M., & Harbor, J. (2009). Exploring the effectiveness of an interdisciplinary water resources engineering module in an eighth grade science course. *International Journal of Engineering Education*, *25*(1), 181.

Robinson, A., Dailey, D., Hughes, G., & Cotabish, A. (2014). The effects of a science-focused STEM intervention on gifted elementary students’ science knowledge and skills. *Journal of Advanced Academics*, *25*(3), 189-213.

Robinson, N. (2016). A Case Study Exploring the Effects of Using an Integrative STEM Curriculum on Eighth Grade Students? Performance and Engagement in the Mathematics Classroom.

Ruiz-Primo, M. A., Briggs, D., Iverson, H., Talbot, R., & Shepard, L. A. (2011). Impact of undergraduate science course innovations on learning. *science*, *331*(6022), 1269-1270.

Sarican, G., & Akgunduz, D. (2018). The Impact of Integrated STEM Education on Academic Achievement, Reflective Thinking Skills towards Problem Solving and Permanence in Learning in Science Education. *Cypriot Journal of Educational Sciences*, *13*(1), 94-107.

Sirajudin, N., & Suratno, J. (2021). Developing creativity through STEM education. Journal of Physics: Conference Series,

Sullivan, F. R. (2008). Robotics and science literacy: Thinking skills, science process skills and systems understanding. *Journal of Research in Science Teaching: The Official Journal of the National Association for Research in Science Teaching*, *45*(3), 373-394.

Sunyoung, H., Rosli, R., Capraro, M. M., & Capraro, R. M. (2016). The effect of science, technology, engineering and mathematics (STEM) project based learning (PBL) on students’ achievement in four mathematics topics. *Journal of Turkish Science Education*, *13*(special), 3.

Tati, T., Firman, H., & Riandi, R. (2017). The effect of STEM learning through the project of designing boat model toward student STEM literacy. Journal of Physics: Conference Series,

Tolliver, E. R. (2016). *The effects of science, technology, engineering and mathematics (STEM) education on elementary student achievement in urban schools* Grand Canyon University].

Toma, R. B., & Greca, I. M. (2018). The effect of integrative STEM instruction on elementary students’ attitudes toward science. *Eurasia Journal of Mathematics, Science and Technology Education*, *14*(4), 1383-1395.

Townes, T. C. (2016). *The consequences of creativity in the classroom: The impact of arts integration on student learning*. Union University.

Wade-Shepherd, A. A. (2016). *The effect of middle school STEM curriculum on science and math achievement scores*.

Yaki, A. A., Saat, R. M., Sathasivam, R. V., & Zulnaidi, H. (2019). Enhancing Science Achievement Utilising an Integrated STEM Approach. *Malaysian Journal of Learning and Instruction*, *16*(1), 181-205.

Yildirim, B., & Selvi, M. (2016). Examination of the effects of STEM education integratedas a part of science technology society and environmentcourses. *Journal of human sciences*, *13*(3).

Yildirim, B., & Selvi, M. (2017). An experimental research on effects of STEM applications and mastery learning. *Journal of Theory and Practice in Education*, *13*(2), 183-210.

Yıldırım, B., & Sidekli, S. (2018). STEM applications in mathematics education: The effect of STEM applications on different dependent variables.
